# The 20^th ^anniversary of EMBnet: 20 years of bioinformatics for the Life Sciences community

**DOI:** 10.1186/1471-2105-10-S6-S1

**Published:** 2009-06-16

**Authors:** Domenica D'Elia, Andreas Gisel, Nils-Einar Eriksson, Sophia Kossida, Kimmo Mattila, Lubos Klucar, Erik Bongcam-Rudloff

**Affiliations:** 1 Institute for Biomedical Technologies, CNR, Via Amendola 122/D, 70126 Bari, Italy; 2 Uppsala Biomedical Centre (BMC), Computing Department, University of Uppsala, Box 570 SE-751 23 Uppsala, Sweden; 3 Bioinformatics & Medical Informatics Team, Biomedical Research Foundation of the Academy of Athens, 11527 Athens, Greece; 4 CSC – IT Center for Science Ltd., Keilaranta 14, 02100 Espoo, Finland; 5 Institute of Molecular Biology, Slovak Academy of Sciences, Dubravska cesta 21, 84551 Bratislava, Slovakia; 6 Department of Animal Breeding and Genetics, Swedish University of Agricultural Sciences, 75024 Uppsala, Sweden

## Abstract

The EMBnet Conference 2008, focusing on 'Leading Applications and Technologies in Bioinformatics', was organized by the European Molecular Biology network (EMBnet) to celebrate its 20^th ^anniversary. Since its foundation in 1988, EMBnet has been working to promote collaborative development of bioinformatics services and tools to serve the European community of molecular biology laboratories. This conference was the first meeting organized by the network that was open to the international scientific community outside EMBnet. The conference covered a broad range of research topics in bioinformatics with a main focus on new achievements and trends in emerging technologies supporting genomics, transcriptomics and proteomics analyses such as high-throughput sequencing and data managing, text and data-mining, ontologies and Grid technologies. Papers selected for publication, in this supplement to *BMC Bioinformatics*, cover a broad range of the topics treated, providing also an overview of the main bioinformatics research fields that the EMBnet community is involved in.

## EMBnet history, mission and activities

EMBnet was established in 1988 as an initiative of the European Molecular Biology Laboratory (EMBL) [1]. At that time, high-speed data communication across Europe as well as bioinformatics were in their infancy. The EMBL was accumulating an ever-growing quantity of sequence data and both the molecular biology and the biotechnology research communities needed to have an easy and fast access to those data. Accessing a remote computer using an ordinary command-line oriented terminal was not enough. The solution for eliminating communication delays could only be found in a distribution of data and computer resources across a number of European nodal centres each one serving its own local research community. The first practical steps were taken by EMBL in the spring of 1988 after getting a positive feedback from scientists around Europe. This idea found the acceptance of most representative computer and research centres, at that time, dealing with molecular data management and analysis. A common agreement was reached in constituting the European Molecular Biology network, the EMBnet [2].

In July 1988, the first EMBnet Workshop was organized at EMBL with participants from EMBL, Daresbury (UK), CITI2 (France), CAOS/CAMM Centre (the Netherlands) and Hoffmann-La Roche. An early focus was on network protocols for the distribution of data from the EMBL Data Library. At first DECNET was intended as the data carrier but it was soon replaced by TCP/IP. A set of client-server data transfer programs, xNDT, was later developed at the Swedish node. Another important issue in the agenda was to apply for a grant for a pilot EMBnet project to the European Community.

In November 1988, a letter was sent from the EMBL Director General to all EMBL Council members asking them to stimulate processes in their regions to identify regional EMBnet nodes. As a proof of the urgent need, at the EMBnet Workshop in May 1989, organized at the EMBL, all 14 EMBL member states and established national nodes were represented, including France, Sweden, the UK, the Netherlands, Spain, Israel, Norway, Italy and Denmark. Switzerland, West Germany, Austria, Greece and Finland were gearing up. In 1991, EMBnet received its first grant from the European Community within its framework BRIDGE (Biotechnology Research for Innovation, Development and Growth in Europe 1990–1994). The major objective of the project was essentially the promotion of EMBnet as a European computer network for bioinformatics. The main topics for the development of the network were essentially three: a) the setting up of a bulletin board, b) the study and development of the technical tools for data distribution and c) the planning of specialised courses and workshops.

A Steering Committee (SC) was nominated during the business meeting held in Nijmegen (NL) in July 1992, the role of which was to promote new projects and to stimulate inter-node cooperation. This and subsequent grants have been important for the successful growth of EMBnet. The initially intended goal of EMBnet was fulfilled. During its first 8 years, the national nodes were the centres where researchers in each European country could access bioinformatics data that were kept in perfect synchrony with the central data repositories at EMBL and its corresponding agencies NCBI at NIH in the USA and DDBJ in Japan.

In 1996, EMBnet was already composed of 26 nodes throughout Europe with a consolidated background in spreading data, computer resources and teaching/training activities serving its research community [3]. The hot topic, at that time, was no more how to distribute data and resources but how to cope with the problem of exploiting, in the best way the huge amount of biological and molecular data collected in primary and specialized databases. There was a great need to link these databases for data integration. The first initiatives in this direction were taken by the NCBI with the development of the Entrez software [4] and by the EMBL in Heidelberg with the development of the SRS (Sequence Retrieval System) software by Thure Etzold [5]. The EMBnet community developed for this system a WWW interface which was installed at each node and which is still one of the most used services by the EMBnet research community [6]. Along with the development of the SRS WWW interface, many others successful initiatives were accomplished. In the year 2000 EMBnet was the promoter in the creation of the peer reviewed journal *Briefings in Bioinformatics *(BiB). BiB was also supported by an educational grant from EMBnet.

The role of EMBnet as a provider of bioinformatics services and expertise for a large community of researchers is underlined by a large series of success stories. With this mission EMBnet has supported the growth of bioinformatics and computational biology in many countries, aiding at the implementation of bioinformatics infrastructures and in the development and usage of the most advanced bioinformatics tools and resources. From very early on, EMBnet has promoted the development of distributed computing services such as HASSLE [7], SRSfed [8] and MRSfed [9] to share workload among international servers and also contributed to the development and maintenance of advanced database systems (Bioimage [10], CpGisle [11], CLEANUP [12], Webin & Seqin [13], GQserv [14], PRINTS [15], InterPro [16], STACKdb [17], UniProt [18], MNyHITS [19], ENSEMBL [20], MitoDrome [21], YeastBASE [22], MRS [23], MitoRes [24], p53FamTag [25], engineDB [26]). EMBnet is also committed to bringing the latest software algorithms to the user, free of charge (EGCG [27], Pratt [28], GeneDoc [29]) and continues to develop state of the art public software such as EMBOSS [30] and powerful, easy to use intuitive interfaces (CINEMA [31], W2H [32], GeneDoc [29], WWW2GCG [33], Jalview [34], wEMBOSS [35], Jemboss [36], STACKpack [17], EMBOSSrunner [37], eBiotools [38], WebLab [39], UTOPIA [40]).

EMBnet has provided major contributions to supercomputing as a means to deliver more powerful and advanced services (Bioccelerator [41], MPSRCH [42], INSECTS and MOLLUSCS [43]) and pioneered the use of Grid technologies for Life Sciences. It has been involved in seminal European Grid projects such as SweGrid [44], EGEE [45], EMBRACE [46], HealthGrid [47], Bioinfogrid [48], and has also developed the first complete e-learning system for teaching bioinformatics (EMBER [49]). Recently a web-based e-learning system [50] has been added to its list of services. The new system is based on the Moodle [51] software with a few plug-in extensions and provides facilities to support on-site training. The e-Learning server is offered as a community service providing training material and experience for end-users, such as bioinformaticians, teachers and researchers. To facilitate sharing/exchange of teaching material, the e-learning web site also provides an exchange service, where the community may share documents, presentations and experiences in bioinformatics training.

Attracted by the high level support of EMBnet, many countries from Asia, Africa and America have joined EMBnet within the last few years, such as Sri Lanka, Pakistan, Kenya and Costa Rica. Any research group willing to take over a certain level of support, or already offering support to a regional community providing tools and databanks in bioinformatics, or which is proficient in the development of such tools or databanks, can apply for the status of National or Associated (Specialist or Industrial) EMBnet node. This has allowed EMBnet to expand well beyond the European frontiers. Currently EMBnet bridges cooperation among 39 member nodes extending to over 31 countries all over the World and reaching thousands of users. In addition, EMBnet also maintains a fruitful cooperation with the Iberoamerican (RIBIO) [52] and the Asia Pacific (APBioNet) [53] bioinformatics networks as well as with the US based International Society for Computational Biology (ISCB) [54]. Close contacts have been established some years ago with the African Society for Bioinformatics and Computational Biology (ASBCB) [55] and fruitful cooperation with other scientific groups in northern Africa are on their way to be realized.

National EMBnet nodes provide local training and support programmes in local languages and also provide their national scientific communities with access to high performance computing resources, specialized databanks and up-to-date software. Some nodes act as redistribution centres to national research institutes and collaborative technical expertise within EMBnet provides support for sustaining the bio-computing facilities of the member nodes.

The quarterly newsletter of EMBnet, the 'EMBnet.news' [56], represents the main interface of the network to its user community presenting reports about its internal activities and latest achievements, together with technical and scientific papers on new developments regarding bioinformatics, computational biology and bio-computing. The recent EMBnet.news issues have greatly increased in size as well as in content, collecting contributions also from their associated communities of partners in European and other national bioinformatics projects. The number of issues downloaded per month amounts to thousands of accesses; indicating a high interest in this news letter outside the EMBnet community and in its role as a reference point for the worldwide bioinformatics community.

## The EMBnet Conference 2008

To celebrate the 20^th ^anniversary of its activity, EMBnet organized, in conjunction with the Annual General Meeting of the network, an international conference on bioinformatics and computational biology [57]. The conference took place in Italy, at the Park Hotel San Michele in Martina Franca (TA) from September 18^th ^to 20^th ^in 2008. The event, called 'Leading Applications and Technologies in Bioinformatics', brought together more than 120 scientists from all over the world to present and discuss new technologies and instruments developed across a vast range of research topics. In addition to the major representation of EMBnet members, the conference saw the contributions of many European researchers (76%) as well as of researchers from America (11%), Asia (8%), Africa (4%) and Australia (1%). Through this conference, EMBnet intended to give an opportunity for aggregation of the worldwide scientific community around the major themes at the frontiers of bio-computing and biological research. The scientific programme was divided into four sessions and the themes covered were 'omics', as the most conventional up-to-date application of bioinformatics and computational biology to Life Sciences research; advanced bioinformatics technologies and applications, such as new technologies for high-throughput sequencing, data- and text-mining instruments for biological research, ontologies, GRID technologies and web services; biodiversity and metagenomics, giving an insight into new trends of bioinformatics in these research areas. Last but not least, a session on training and e-Learning in Bioinformatics was held, which covered the educational aspects within Bioinformatics to keep end users updated with the latest developments implementing technologies for e-Learning and other instruments and to share their 'pros and cons' amongst developers as well. Presentations in this area were then the topics of an afternoon round table discussion during which many of the instruments and experiences presented at the morning conference session were demonstrated and further discussed.

The conference was opened by the conference chair Domenica D'Elia, node manager of the Italian EMBnet National node and representative of the hosting institute, the CNR Institute for Biomedical Technologies in Bari. The conference opening ceremony included the celebration of the EMBnet 20^th ^anniversary with two presentations. Firstly Prof. Cecilia Saccone, as one of the first and major promoters of EMBnet, reminded us about the EMBnet history, its aim and missions. She explained that the goals have not changed much since the 80's, apart from an opening to the rest of the world. We should continue to expand the network, touch new fields for teaching (interdisciplinary) and develop research. The second celebrative talk was presented by associate professor Erik Bongcam-Rudloff (EMBnet chairman) who gave a demonstrative view of the future of EMBnet with a shuffled movie to illustrate new challenges for bioinformatics research that we are going to deal with, such as the huge amount of data coming from high-throughput technologies and applications. He also initiated a discussion concerning a new name for EMBnet which should reflect the evolution of the network from an European dimension to the new worldwide expansion, also reflecting the links to other collaborating networks such as RIBio, APBioNet and ASBCB.

The scientific sessions of the conference were opened by the keynote speakers Mehrdad Hajibabaei (Biodiversity Institute of Ontario, University of Guelph Canada), Indra Neil Sarkar (MBLWHOI Library, Marine Biological Laboratory, USA), Tin Wee Tan (Department of Biochemistry, YLL School of Medicine, National University of Singapore), Alexander E. Kel (BIOBASE GmbH, Germany) and Vincent Breton (Université Blaise Pascal, Clermont-Ferrand – France) and included 31 selected speakers among the 76 who had submitted abstracts for presentation at the conference. A complete report about the conference, by the EMBnet Executive Board secretary Laurent Falquet, has been published on EMBnet.news [58].

A tutorial on 'Grid Computing' was organized as a satellite conference event on September 17th. The tutorial was organized thanks to a joint effort of EMBnet and the LIBI Italian FIRB project [59]. It was aimed at research students, post-docs, and senior researchers with an interest in using or developing applications for distributed computing environments [60]. More than 30 participants and 12 teachers attended the tutorial. After an introductory talk by Josè R. Valverde, from the Spanish EMBnet Node (Centro Nacional de Biotecnología in Madrid), the tutorial focused on presentations, hands-on and demos on some of the tools recently developed inside the LIBI project. Particular emphasis was given to the GRID Problem Solving Environment developed and set up for the LIBI project, the bioinformatics grid portals enabled with robot certificates, the LIBI federated databases approach and the tools used for accessing it from a GRID environment. Some examples of bioinformatics workflow executions on their platform through high-level workflow management tools like Taverna [61], were also presented. Articles from tutorial presentations have been published on the EMBnet.news issue 14.4.

The conference programme, abstracts selected as oral and poster presentations along with some informative articles on EMBnet history and activities have been published in the EMBnet.news issue 14.3.

## Review policy

From the 76 abstracts submitted to the conference, 31 were selected for oral presentation. The remaining selected abstracts were presented in poster conference sessions. Papers submitted to these proceedings were peer-reviewed by at least two reviewers from the scientific committee board of the EMBnet Conference and by external experts as required, in total 33 reviewers (see Additional file 1). To manage the whole reviewing process we used a fully automated on-line system, the Open Conference System from the Public Knowledge Project [62]. We received 29 full papers, however following our reviewing policy we could accept only 24, selected on the basis of various criteria such as scientific and technical relevance as well as novelty of the approach and relevance of results presented. This supplement to *BMC Bioinformatics *features these 24 papers, which reflect the character of the conference and its focus on emerging research fields in bioinformatics and computational biology.

## An overview of proceeding contents

### Genomics data analysis

With multiple genome sequences publicly available, we are now in the (post-) genomics era. The so called Next generation (NextGen), rapid, low-cost sequencing techniques is making it possible to address a broad range of genetic analysis applications including: comparative genomics, high-throughput polymorphism detection, analysis of transcriptional regulation, unraveling mutant genes in diseases, and many other studies, only limited by the researchers imagination.

Comparative genomics is a central step in many sequence analysis studies and the annotation of whole genomes through the identification of coding and regulatory regions is one of the major challenges in the current research in molecular biology. Creanza et al. [63] present a statistical assessment of discriminative features for protein-coding and non coding cross-species conserved sequence elements, comparing distributions of a set of comparative and non comparative features and evaluating the prediction accuracy of classifiers trained for discriminating sequence elements conserved among human, mouse and rat species.

Penel et al. [64], developed an automated procedure allowing massive all-against-all similarity searches, gene clustering, multiple alignments computation, and phylogenetic tree reconstruction and reconciliation which led to the production of three databases: HOVERGEN, HOGENOM and HOMOLENS.

Other example of new tools are represented by the work of Sperber et al. [65], who created 'RetroTector' for the study of retroviral elements in vertebrates; by the work of Rubino and Attimonelli [66], who present a new algorithm for the classification of sequences based on regular expression syntax; and by the work of Sebestyén et al. [67] presenting DoOPSearch, a web-based tool using the comparative analysis of a large number of orthologous promoter regions to find common conserved motifs in the promoter regions of different chordate and plants genes, and to identify the overrepresented Gene Ontology terms for functional gene correlations.

Calderon-Copete et al. [68] and Zhe Li et al. [69] give examples of the broad spectrum of emerging new genomes presenting works on Mycoplasma and plant respectively.

### Transcriptomics data analysis

The assessment of the functional aspects of time-course transcriptomics data requires the use of approaches that exploit the activation dynamics of the functional categories to where genes are annotated to. A new complex approach in the evaluation of the expression data is presented by Nueda et al. [70]. In this work authors present three new methods able to capture different aspects of the relationship between genes, functions and coexpression that are biologically meaningful. Another interesting aspect of large-scale transcriptome data analysis is treated by Picardi et al. [71] with EasyCluster, a new clustering tool able to generate gene-oriented clusters of ESTs when a genomic sequence and a pool of related expressed sequences are provided.

### Proteomics data analysis

Two key elements of omics are automatic data analysis and data visualization. Moschopoulos et al. [72] present a new clustering tool called GIBA and demonstrate how combining existing methods, in this case clustering tools used to analyze protein-protein interactions, can increase the quality of the results. Tsagrasoulis et al. [73] describe a visualization tool to compare two protein LC-MS datasets at a very detailed level; while Strömbergsson and Kleywegt [74] present a new computational approach to visualize and compare chemogenomics protein-ligand subspaces.

### Molecular biodiversity and DNA barcode

DNA sequences have become a primary source of information in biodiversity analysis. Singer and Hajibabaei [75] present a web-based toolkit which allows the user to manage their barcode datasets, pull out non-unique sequences, identify haplotypes within a species, and examine the within- to between-species divergences. In addition, they provide a number of phylogenetics tools that will allow the user to manipulate phylogenetic trees generated by other popular programs.

A standardized and cost-effective molecular identification system for Fungi is an urgent need owing to their wide involvement in human life quality. However, mobile introns in almost all the fungal mitochondrial genes represent a serious difficulty in PCR and bioinformatics surveys. Santamaria et al. [76] developed a query-based approach searching in public databases for those mobile introns and compare the results with a BLAST-based approach.

### Systems biology

The article from Gerdtzen et al. [77] presents a mathematical model based on the gene network involved in heterocyst differentiation and depicts a good attempt towards the systems biology approach. Another valuable attempt in this direction, i.e. the holistic understanding of biology, is given by the article from Picard et al. [78], which presents MixNet, a software that analyzes biological networks using mixture models.

### Biological data integration

Integration of automatic prediction results and genomic visualization for analysis of genome data is a big issue of the post-genomic era. Barrio et al. [79] describe their work on annotation and visualization of endogenous retroviral sequences using the Distributed Annotation System (DAS) and eBioX. Pettifer et al. [80] apply experience in human-computer interaction (HCI), high-performance rendering and distributed systems to build reusable software components that, together, create a toolkit that is both architecturally sound from a computing point of view, and addresses both user and developer requirements for large-scale analyses which require drawing together data from a variety of geographically and structurally different databases.

Roubelakis et al. [81] present GOmir, a novel stand-alone application consisting of two separate tools for the analysis of microRNAs (miRNAs) target genes: JTarget and TAGGO.

### Grid technologies and web services

Two interesting pieces of work how the Grid technology is used, are represented by the article from Barbera et al. [82], describing the GENIUS Grid Portal and the robot certificates; and by the article from Minervini et al. [83], that describe massive non natural proteins structure prediction using the Grid. The former deals with the Grid technology itself, whereas the latter presents an application.

### Data and text mining

Data and text mining techniques represent two important IT applications for biological knowledge discovery. Castellano et al. [84] developed a software middleware solution in order to exploit the many knowledge discovery software applications on scalable and distributed computing systems, such as the GRID infrastructure, to tackle the intensive use of information and communication resources in Life Sciences. Lagani et al. [85] describe a new kernel function consisting of a similarity measure between groups of subjects genotyped for numerous genetic loci. Turi et al. [86] present UTRminer, a new application of data mining techniques for the discovery of *cis-regulatory *modules controlling translation of mRNAs targeting the mitochondrion.

## Competing interests

The authors declare that they have no competing interests.

## Supplementary Material

Additional file 1 Review Committee members of EMBnet Conference 2008 proceedings. Click here for file
