# Supplementary material for: The 20th anniversary of EMBnet: 20 years of bioinformatics for the Life Sciences community
Source: BMC Bioinformatics. 2009 Jun 16;10(Suppl 6):S1. doi: 10.1186/1471-2105-10-S6-S1 (PMC2697632; doi:10.1186/1471-2105-10-S6-S1)
Supplement: Additional file 1 — Review Committee members of EMBnet Conference 2008 proceedings. [file 1471-2105-10-S6-S1-S1.pdf]

	Name	Surname	Affiliation
1	Teresa	Attwood	Faculty of Life Sciences, Manchester University, Manchester, United Kingdom
2	Emiliano	Barreto	Bioinformatics Center, Biotechnology Institute, National University of Colombia, Bogotá, Colombia
3	Endre	Barta	Bioinformatics Group, Agricultural Biotechnology Center, Gödöllo, Hungary
4	Erik	Bongcam-Rudloff	Department of Animal Breeding and Genetics, Swedish University of Agricultural Sciences, Biomedical centre, Uppsala, Sweden
5	Shahid	Chohan	Dept. of Biosciences, COMSATS Institute of Information Technology, Islamabad, Pakistan
6	Domenica	D'Elia	Institute for Biomedical Technologies, CNR, Bari, Italy
7	Laurent	Falquet	Swiss Institute of Bioinformatics, Lausanne, Switzerland
8	Pedro	Fernandes	Instituto Gulbenkian de Ciencia, Centro Portugues de Bioinformatica, Oeiras, Portugal
9	Andreas	Gisel	Institute for Biomedical Technologies, CNR, Bari, Italy
10	Mehrdad	Hajibabaei	Canadian Centre for BNA Barcoding, Biodiversity Institute of Ontario, University of Guelph, Guelph, Canada
11	Jagger	Harvey	Biosciences eastern and central Africa (BecA) Hub, Plant Biosciences Research Scientist, Nairobi, KENYA
12	Sophia	Kossida	Academy of Athens, Biomedical Research Foundation, Athens, Greece
13	Jack A.M.	Leunissen	Netherlands Bioinformatics Centre, NBIC,Nijmegen, The Netherlands
14	Jingchu	Luo	College of Life Sciences, Peking University, Beijing, China
15	George	Magklaras	The Biotechnology Centre of Oslo, The University of Oslo, Biotek - UiO Oslo, Norway
16	Christos	Makris	Computer Engineering and Informatics Department, University of Patras, Patras, Greece
17	Kimmo	Mattila	CSC — IT Center for Science Ltd., Espoo, Finland
18	Georgios	Papachristoudis	Massachusetts Institute of Technology, Cambridge, MA, USA
19	Ricardo Bringas	Perez	Centro de Ingenieria Genetica y Biotecnologia,La Habana, Cuba
20	Guy	Perriere	Laboratoire de Biométrie et Biologie Evolutive, University of Lyon, Villeurbanne, France
21	Graziano	Pesole	1. Department of Biochemistry and Molecular Biology, University of Bari, Bari, Italy;<br>2. Institute for Biomedical Technologies, CNR, Bari, Italy
22	Sandor	Pongor	International Centre for Genetic Engineering and Biotechnology, AREA Science Park,Trieste, Italy
23	Fotis	Psomopoulos	Aristotle University of Thessaloniki,Department of Electrical and Computer Engineering, Thessaloniki, Greece
24	Shoba	Ranganathan	1. Department of Chemistry and Biomolecular Sciences and ARC Centre of Excellence in Bioinformatics, Macquarie University, Sydney, Australia;<br>2. Department of Biochemistry, Yong Loo Lin School of Medicine, National University of Singapore, Singapore
25	Cecilia	Saccone	1. Dipartimento di Biochimica e Biologia Molecolare, Università di Bari, Bari, Italy;<br>2. Institute for Biomedical Tecnologies, CNR, Bari, Italy
26	J. Cristian	Salgado	Centre for Biochemical Engineering and Biotechnology, Department of Chemical Engineering and Biotechnology, University of Chile, Santiago, RCH
27	Sebastiano	Stramaglia	Department of Physics , University of Bari, Italy
28	Evangelos	Theodoridis	Computer Engineering and Informatics Department, University of Patras, Patras, Greece
29	Angelica	Tulipano	Institute for Biomedical Technologies, CNR, Bari, Italy
30	Josè Ramon	Valverde	Scientific Computing Service, Madrid, Spain
31	Ana Tereza	Vasconcelos	Laboratório Nacional de Computação Científica,Laboratório de Bioinformática, Quitandinha Petrópolis, Rio de Janeir, Brazil
32	Saverio	Vicario	Institute for Biomedical Technologies, CNR, Bari, Italy
33	Piotr	Zielenkiwicz	Institute of Biochemistry and Biophysics, Polish Academy of Sciences, Warszawa, Poland
